# Pilot Biomarker Analysis and Decision Tree Algorithm Modeling of Patients with Chronic Subdural Hematomas

**DOI:** 10.1089/neur.2022.0062

**Published:** 2023-03-24

**Authors:** David J. Puccio, Hansen Deng, Shawn R. Eagle, David O. Okonkwo, Enyinna L. Nwachuku

**Affiliations:** Department of Neurological Surgery, University of Pittsburgh Medical Center, Pittsburgh, Pennsylvania, USA.

**Keywords:** biomarker, chronic subdural hematoma, CT imaging, Glasgow Outcome Scale-Extended, inflammation, traumatic brain injury

## Abstract

The elderly population are at high risk for developing chronic subdural hematoma (cSDH). Surgical evacuation of cSDH is one of the most common procedures performed in neurosurgery. The present study aims to identify potential inflammatory biomarkers associated with its development and recurrence. Patients (>65 years of age) who presented with symptomatic cSDH (≥1 cm thickness or ≥5 mm midline shift [MLS]), requiring surgical intervention, were prospectively enrolled. The collected cSDH fluid was analyzed for inflammatory markers. Computed tomography (CT) scan data included pre-operative cSDH thickness and MLS. Outcome data included Glasgow Outcome Scale-Extended (GOS-E) score at 3, 6, and 12 months post-surgery, as well as cSDH recurrence. A decision tree model was used to determine the predictive power of extracted analytes for MLS, cSDH thickness, and recurrence. This pilot study includes 20 enrolled patients (mean age 77.9 ± 7.4 years and 85% falls). Rate of cSDH recurrence was 42%, with 21% requiring reoperation. Chemokine (C-X-C motif) ligand 9 (CXCL9) concentrations correlated with cSDH thickness (*r* = 0.975, *p* = 0.040). Interleukin (IL)-6 and vascular endothelial growth factor (VEGF)-A concentrations correlated with MLS (*r* = 0.974, *p* = 0.005; *r* = 0.472, *p* = 0.036, respectively). IL-5 concentrations correlated with more favorable GOS-E scores at 3, 6, and 12 months (*r* = 0.639, *p* = 0.006; *r* = 0.727, *p* = 0.003; *r* = 0.693, *p* = 0.026, respectively). Regulated on activation, normal T-cell expressed and secreted (RANTES) concentrations correlated with complete cSDH resolution (*r* = 0.514, *p* = 0.021). The decision tree model identified that higher concentrations of CXCL9 were predictive of MLS (risk ratio [RR] = 12.0), higher concentrations of IL-5 were predictive of cSDH thickness (RR = 4.5), and lower concentrations of RANTES were predictive of cSDH recurrence (RR = 2.2). CXCL9, IL-6, VEGF, IL-5, and RANTES are associated with recurrence after surgery and may be potential biomarkers for predicting cSDH recurrence and neurological outcomes.

## Introduction

Chronic subdural hematoma (cSDH) is a common disease in an aging population.^1^ The annual incidence rate ranges from 1.72 to 20.6 per 100,000 persons and continues to rise globally.^2–4^ In a study conducted in Japan, incidence increased from 13.1 to 20.6 per 100,000 persons from 1986 to 2007, similar to findings in the Finnish population.^5–7^ Elderly patients are more prone to brain atrophy and vascular fragility, greater fall risk, as well as usage of antiplatelets and -coagulants.^8–10^ In the United States, cSDH evacuation is one of the most common neurosurgical procedures performed, with significant financial burden on the current healthcare system.^11–13^

Subsequent to traumatic brain injury (TBI), cSDH can initiate an inflammatory response from dural border cells that leads to the formation of neomembranes and leaky capillaries. This propagates a sustained inflammatory response with continuous blood and fluid collection.^14^ Injury of the middle meningeal artery (MMA), which supplies these capillaries, could cause additional bleeding risk into the subdural space.^15,16^ cSDH is generally hypodense on computed tomography (CT),^17^ and asymptomatic patients are managed conservatively with observation.^18^ For symptomatic patients requiring surgical treatment, evidence shows improved outcomes with a subdural drain placement, with some variability in the surgical techniques of burr hole craniostomy, craniotomy, or twist-drill craniostomy performed from one institution to another.^19–21^

Mortality rate ranges from 2.7% to 4.6%, and an estimated 5–33% of patients have recurrent cSDH requiring repeated surgeries.^22,23^ Ducruet and colleagues reported higher recurrence rates after twist-drill craniostomy (28.1%) compared to craniotomy (19.4%) and burr hole craniostomy (11.7%).^24^ Management of recurrent cSDH remains a challenge to the clinician because this pathology is associated with increased hospital stay, morbidity, and mortality in a high-risk population.^11^

An active field of research on cSDH is the exploration of peripheral or local biomarkers to guide the prediction of patient outcomes and risk of recurrence. Past research suggests that there is an inflammatory drive in its formation.^14^ However, the complete mechanistic pathway is yet to be elucidated. Pro- and anti-inflammatory biomarkers have the potential to aid clinical decision making regarding diagnosis, prognosis, and providing markers by which to target treatments. Predictive models that utilize concentrations of peripheral or local biomarkers have yet to be performed. By recruiting persons undergoing cSDH evacuation, the purpose of this pilot study is to provide proof of concept that serum, plasma, and/or intraoperative specimens can be useful for identifying prognostic biomarkers of post-surgical cSDH recurrence.

## Methods and Materials

### Study design and enrollment

Each participant was prospectively enrolled under an institutional review board–approved protocol. Patients who underwent cSDH evacuation at the University of Pittsburgh Medical Center from September 2019 to September 2020 were enrolled in the Brain Trauma Research Center (BTRC). Inclusion criteria included the following: 1) >65 years of age; 2) CT scan consistent with a diagnosis of cSDH with >/ = 10 mm thickness and/or >/ = 5 mm midline shift (MLS); 3) intent to undergo surgical intervention for cSDH with collection of subdural fluid; and 4) signed informed consent from a legal authorized representative. Patients with the following medical conditions were excluded: 1) active malignancy or medical condition which in the opinion of the investigator would compromise analysis; 2) neurodegenerative disease (including, but not limited to, Parkinson's disease, Huntington's disease, Alzheimer's disease, and dementia); 3) debilitating neuropsychiatric disease (including, but not limited to, schizophrenia and bipolar disorder); or 4) past medical history of a chronic immunosuppressive state (HIV/AIDS, transplantation, lupus, or rheumatoid arthritis). Patients were excluded if their cSDH was iatrogenic (i.e., post-lumbar puncture, etc.) or if they were pregnant.

Enrolled patients were taken to the operating room for surgical evacuation, and the specimens were collected under sterile conditions. Surgical techniques included a minicraniotomy or through burr hole craniostomy with subdural drain placement. Serum and plasma were collected perioperatively from an existing vascular access site or by venipuncture.

### Biospecimen analysis

Collected samples were stored within the biorepository system at the BTRC. They were centrifuged and stored in 0.5-mm aliquots at −80°C for future batch analyses. Samples were analyzed by a central, independent laboratory at the Hillman Cancer Center using a Luminex assay, specifically, MILLIPLEX^®^ Human Cytokine/Chemokine/Growth Factor Panel A, which was a broad array including 48 analytes (pg/mL) for inflammatory markers, with the capability to calculate concentrations for each biomarker in subdural fluid samples, as used previously in our laboratory.

### Patient and outcome variables

Demographic and clinical data were collected by research nurse coordinators, including patient age, sex, mechanism of injury, admission symptoms and neurological status (Glasgow Coma Scale [GCS] score), and use of antiplatelets and -coagulants. All study patients were followed post-operatively and were assessed by the primary neurological outcome Glasgow Outcome Scale–Extended (GOS-E) at 3, 6, and 12 months as well as secondary neurological outcomes, including mortality, cSDH recurrence, and other complications.

Radiographical findings were collected and analyzed by a trained neurosurgeon. Patient CT scans were examined before surgery to ascertain pathology, MLS (mm), and thickness of the subdural hematoma (mm). Magnetic resonance imaging (MRI) T1-weighted image (T1WI) scans were also obtained before surgery in order to classify each cSDH as hyperdense, hypodense, isodense, or mixed-density collections. Along with post-operative clinical examinations, immediate post-evacuation CT imaging and at follow-up were analyzed. Determination of the complete resolution of cSDH after evacuation was based on a formal evaluation by a board-certified neuroradiologist.

### Statistical analysis

Descriptive statistics for continuous variables are reported as means and standard deviations (SDs) and, for categorical variables, are reported as percentages. Pearson correlations for continuous variables and Spearman correlations for ordinal variables were performed. Mean concentrations and SDs of each biomarker measured in the subdural fluid are reported in the supplementary material (Supplementary Table S1). A minimum mean threshold value of 1000 pg/mL was selected as inclusion criteria, yielding 17 biomarkers for the present analysis. These analyses were performed using the Statistical Package for Social Sciences (SPSS; version 28; IBM Corporation, Chicago, IL). The C5.1 algorithm was used to identify blood biomarkers associated with cSDH recurrence. C5.1 is a data-mining algorithm that can incorporate continuous, binary, ordinal, and nominal data to create a decision tree as an analytical method for differentiating the outcome (i.e., cSDH recurrence) by maximizing information gain. The resulting decision tree should be considered preliminary and not yet suitable for clinical use.

The decision tree is built such that the strongest predictor appears at the first “split” and continues iteratively down the tree until no further splits contribute to information gain. *Post hoc* relative risk ratios (relative risk; RR) and 95% confidence intervals (CIs) were conducted to identify the risk of the predictor at the algorithm-identified cut point for the outcome. Binary variables were created for each cut point, and univariate logistic regression models were built to obtain the percent variance (*R*^2^) of the most important predictor from each model to the outcome. Only subdural fluid biomarkers were included as potential predictors in the decision tree. Three decision tree models were built, predicting three different outcomes: 1) cSDH recurrence; 2) MLS; and 3) cSDH thickness. cSDH recurrence was the primary outcome, but midline shift and hematoma size were included because previous research has repeatedly demonstrated that they are strong predictors of recurrence.^25,26^ This analysis was performed using SPSS Modeler software (v 18.1).

## Results

### Demographic and clinical presentation

Patient demographic, clinical, and radiographical characteristics are summarized below (Table 1). Between September 2019 and September 2020, a total of 22 study patients were enrolled. Two patients were excluded from the current analysis because of incomplete biomarker sets to yield 20 patients. Average age was 77.9 (±7.4) years and predominantly male (*N* = 13; 65%). All patients presented with a GCS score of 15, and the mechanism of injury in 17 patients (85%) was a fall, whereas 3 patients (15%) presented with no known mechanism. Primary admission symptoms included headache (*N* = 7; 35%), gait disturbance (*N* = 6; 30%), altered mental state (*N* = 3; 15%), speech issues (*N* = 1; 5%), hemiparesis (*N* = 1; 5%), syncope (*N* = 1; 5%), and seizure (*N* = 1; 5%). Anticoagulant and -platelet usage upon admission included none (*N* = 8; 40%), aspirin (*N* = 7; 35%), clopidogrel (*N* = 2; 10%), both aspirin and clopidogrel (*N* = 2; 10%), and apixaban (*N* = 1; 5%).

**Table 1.** Demographic and Clinical Presentation Characteristics

Demographic and clinical presentation characteristics (*N*)	*N*/percentage (%)	Mean (SD)
Age		77.85 (7.37)
Presenting GCS score		15 (0)
Sex		
Male	13/65	
Female	7/35	
Presenting injury		
Fall	17/85	
None	3/15	
Primary admission symptom		
Headache	7/35	
Gait disturbance	6/30	
Altered mental state	3/15	
Speech issues	1/5	
Hemiparesis	1/5	
Syncope	1/5	
Seizure	1/5	
Anticoagulant/antiplatelet		
None	8/40	
Aspirin	7/35	
Plavix	2/10	
Aspirin/Plavix	2/10	
Eliquis	1/5	

SD, standard deviation; GCS, Glasgow Coma Scale.

### Radiographical and intraoperative data

Radiographical and intraoperative data are summarized below (Table 2). By laterality, patients presented with cSDH on the right (*N* = 11; 55%) and left (*N* = 6; 30%) sides of the brain, as well as bilaterally (*N* = 3; 15%). CT findings yielded an average cSDH thickness of 20.0 (±5.9) mm and MLS of 6.4 (±4.1) mm. Pre-operative MRI characteristics included hyperdense (*N* = 6; 30%), hypodense (*N* = 5; 25%), not applicable (N/A; *N* = 5; 25%), isodense (*N* = 2; 10%), and mixed density (*N* = 2; 10%). cSDH was evacuated using the minicraniotomy technique in 85% of patients (*N* = 17) and burr hole craniostomy in 15% of patients (*N* = 3), supplemented by subdural drain placement in 95% of patients (*N* = 19). Complete resolution of cSDH after evacuation was achieved in 15% of patients (*N* = 3).

**Table 2.** Radiographical and Intraoperative Data

Radiographical and intraoperative data	*N*/percentage (%)	Mean (SD)
Side of pathology		
Right	11/55	
Left	6/30	
Bilateral	3/5	
MRI T1WI		
Hyperdense	6/30	
Hypodense	5/25	
N/A	5/25	
Isodense	2/10	
Mixed density	2/10	
cSDH thickness (mm)		19.95 (5.87)
MLS (mm)		6.36 (4.06)
Type of evacuation		
Cookie cutter	17/85	
Burr hole craniostomy	3/15	
Subdural drain placement		
Yes	19/95	
No	1/5	
Complete resolution		
Yes	3/15	
No	17/85	

SD, standard deviation; MRI T1WI, magnetic resonance imaging T1-weighted image; N/A, not applicable; cSDH, chronic subdural hematoma; mm, millimeter; MLS, midline shift; MMA, medial meningeal artery.

### Biomarker levels and outcomes measures

Outcome measures are summarized below (Table 3). In-hospital mortality occurred in 4 patients (20%) and complications occurred in 6 patients (30%), including empyema (*N* = 1), temporal venous stroke (*N* = 1), hypoxic respiratory failure (*N* = 1), post-operative recurrence (*N* = 1), seizures (*N* = 1), and meningitis (*N* = 1). The patient with hypoxic respiratory failure died before post-operative CT imaging was obtained. Of the remaining 19 patients, cSDH recurrence occurred in 8 patients (42%), with 4 patients (21%) requiring reoperation. Median GOS-E scores at 3, 6, and 12 months were 7 (*N* = 17), 7.5 (*N* = 14), and 6.5 (*N* = 10), respectively. Favorable outcome, as defined by a GOS-E score of 4–8, was recorded in 12 of 17 patients at the 3-month time point (70.6%), 9 of 14 recorded patients at 6 months (64.3%), and 5 of 10 patients at 12 months (50.0%).

**Table 3.** Outcome Measures

Outcome measures	*N*/percentage (%)	Median
In-hospital mortality		
Yes	4/20	
No	16/80	
Complication		
Yes	6/70	
No	14/30	
Recurrence requiring reoperation		
Yes	4/21	
No	15/79	
Recurrence without reoperation		
Yes	4/21	
No	14/79	
GOS-E at 3 months		7.0
GOS-E at 6 months		7.5
GOS-E at 12 months		6.5

GOS-E, Glasgow Outcome Scale-Extended.

Of the 48 biomarkers measured by the Luminex assay, 17 met the minimum mean threshold of 1000 pg/mL for the present analysis. Their mean concentrations are reported below (Table 4). Chemokine (C-X-C motif) ligand (CXCL9) had the greatest average concentration (16,484.76 ± 19,752.4 pg/mL), followed by monocyte chemoattractant protein-1 (MCP-1; 12,291.6 ± 5422.6 pg/mL), granulocyte colony-stimulating factor (G-CSF; 11,734.7 ± 18,225.9 pg/mL), interleukin 6 (IL-6; 6967.7 ± 3970.0 pg/mL), vascular endothelial growth factor (VEGF)-A (5166.2 ± 4538.9 pg/mL), interleukin 8 (IL-8; 4206.0 ± 2835.0 pg/mL), platelet-derived growth factor-AB,-BB (PDGF-AB/BB; 4011.3 ± 3934.0 pg/mL), platelet-derived growth factor-AA (PDGF-AA; 3654.6 ± 2578.9 pg/mL), macrophage-derived chemokine (MDC; 2693.4 ± 2026.5 pg/mL), interleukin 1 alpha (IL-1α; 2229.0 ± 6430.0 pg/mL), interferon-γ-inducible protein 10 (IP-10; 2029.4 ± 2968.3 pg/mL), regulated on activation, normal T-cell expressed and secreted (RANTES; 1827.4 ± 3234.2 pg/mL), interleukin 10 (IL-10; 1800.4 ± 5418.4 pg/mL), interleukin 1 beta (IL-1β; 1588.7 ± 5535.3 pg/mL), interleukin 1 receptor antagonist (IL-1Ra; 1518.5 ± 4179.9 pg/mL), interleukin 5 (IL-5; 1359.4 ± 3041.1 pg/mL), and macrophage colony-stimulating factor (M-CSF; 1067.2 ± 1139.8 pg/mL).

**Table 4.** Mean Analyte Data

Analyte	Mean (SD) (pg/mL)
CXCL9	16,484.76 (19,752.39)
MCP-1	12,291.55 (5422.59)
G-CSF	11,734.69 (18,225.92)
IL-6	6967.71 (3969.95)
VEGF-A	5166.21 (4538.88)
IL-8	4206.02 (2834.98)
PDGF-AB BB	4011.25 (3934.01)
PDGF-AA	3654.59 (2578.93)
MDC	2693.38 (2026.49)
IL-1α	2228.97 (6429.97)
IP-10	2029.45 (2968.25)
RANTES	1827.40 (3234.22)
IL-10	1800.45 (5418.43)
IL-1β	1588.65 (5535.27)
IL-1Ra	1518.49 (4179.95)
IL-5	1359.4 (3041.09)
M-CSF	1067.18 (1139.76)

SD, standard deviation; pg, picogram; mL, milliliter; CXCL9, chemokine (C-X-C motif) ligand 9; MCP-1, monocyte chemoattractant protein-1; G-CSF, granulocyte colony-stimulating factor; IL-6, interleukin 6; VEGF-A, vascular endothelial growth factor; IL-8, interleukin 8; PDGF-AB BB, platelet-derived growth factor-AB,-BB; PDGF-AA, platelet-derived growth factor-AA; MDC, macrophage-derived chemokine; IL-1α, interleukin 1 alpha; IP-10, interferon-γ-inducible protein 10; RANTES, regulated on activation, normal T-cell expressed and secreted; IL-10, interleukin 10; IL-1β, interleukin 1 beta; IL-1Ra, interleukin 1 receptor antagonist; IL-5, interleukin 5; M-CSF, macrophage colony-stimulating factor.

**FIG. 1.**
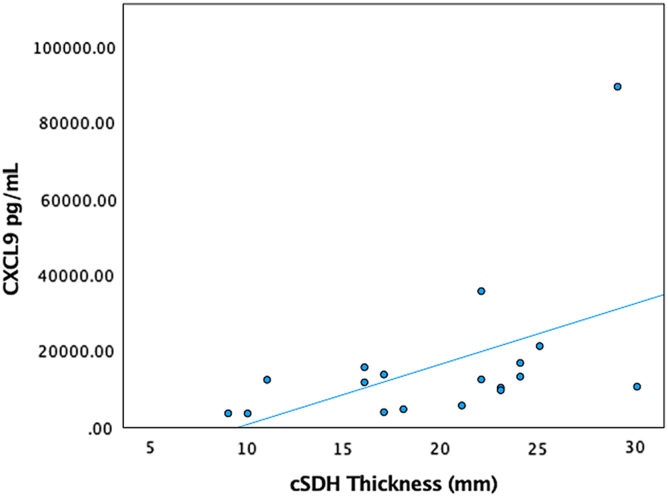
CXCL9 versus cSDH thickness. CXCL9, chemokine (C-X-C motif) ligand 9; cSDH, chronic subdural hematoma; pg, picogram; mL, milliliter; mm, millimeter .

**FIG. 2.**
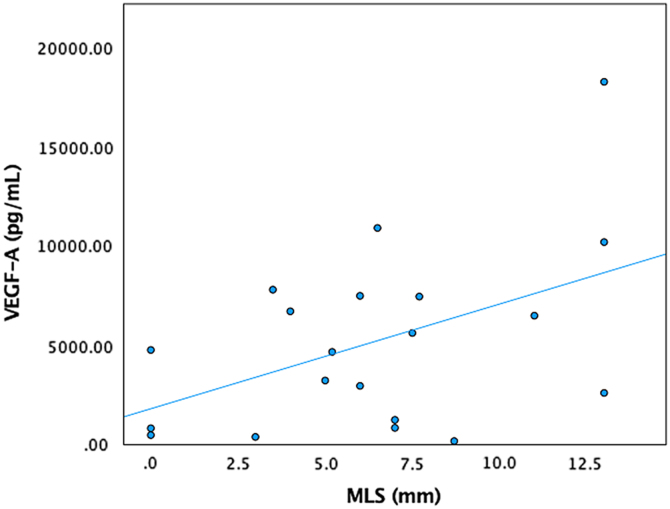
VEGF-A versus MLS. VEGF-A, vascular endothelial growth factor; MLS, midline shift; pg, picogram; mL, milliliter; mm, millimeter.

Higher CXCL9 levels had a very high positive correlation with cSDH thickness on the presenting CT scan (*r* = 0.975, *p* = 0.040; Fig. 1). Higher IL-6 values had a very high positive correlation with MLS (*r* = 0.974, *p* = 0.005). Further, patients with higher VEGF-A levels had a moderately positive correlation with MLS (*r* = 0.472, *p* = 0.036; Fig. 2). Higher IL-5 values had a strong positive correlation with GOS-E at 3, 6, and 12 months (*r* = 0.639, *p* = 0.006; *r* = 0.727, *p* = 0.003; *r* = 0.693, *p* = 0.026). Higher RANTES values had a moderately positive correlation with the complete resolution of cSDH after evacuation (*r* = 0.514, *p* = 0.021).

### Decision tree analysis results

#### Midline shift >10 mm

**FIG. 3.**
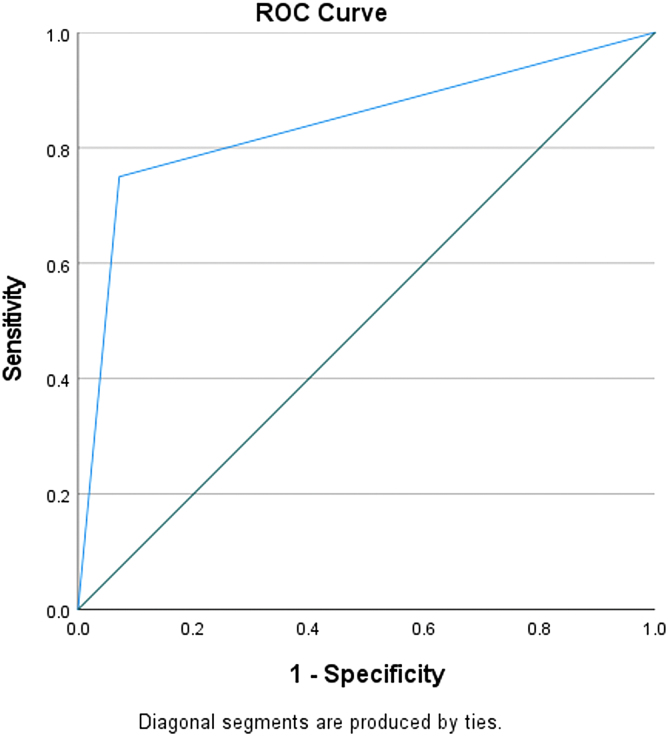
Receiver operating characteristic (ROC) curve of CXCL9 concentrations to discriminate between cSDH patients with midline shift >10 mm. CXCL9, chemokine (C-X-C motif) ligand 9; cSDH, chronic subdural hematoma; mm, millimeter.

**FIG. 4.**
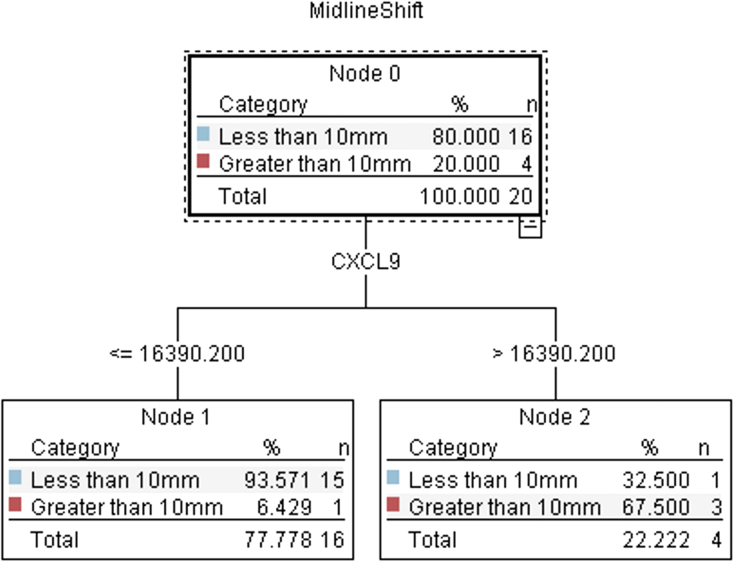
C5.1 decision tree demonstrating that CXCL9 concentrations >16,390.2 pg/mL were significantly associated with midline shift >10 mm in cSDH patients. CXCL9, chemokine (C-X-C motif) ligand 9; mm, millimeter; cSDH, chronic subdural hematoma; pg, picogram; mL, milliliter.

The C5.1 algorithm built a decision tree model with 90% accuracy and an area under the curve (AUC) of 0.83 (Fig. 3). The only significant predictor of an MLS >10 mm was a CXCL9 concentration of 16,390.2 pg/mL (Fig. 4). A CXCL9 concentration above that value was associated with an RR of 12 (95% CI, 1.7–87.0; *p* < 0.001) compared to patients with a CXCL9 concentration below that value. When using the CXCL9 cutoff as a predictor in a univariate logistic regression model of MLS >10 mm, it accounted for 51% of the variance (*R*^2^ = 0.51, *p* = 0.01). An MLS >10 mm on the presenting CT scan has been found to be significantly related to cSDH recurrence.

#### Hematoma thickness >20 mm

**FIG. 5.**
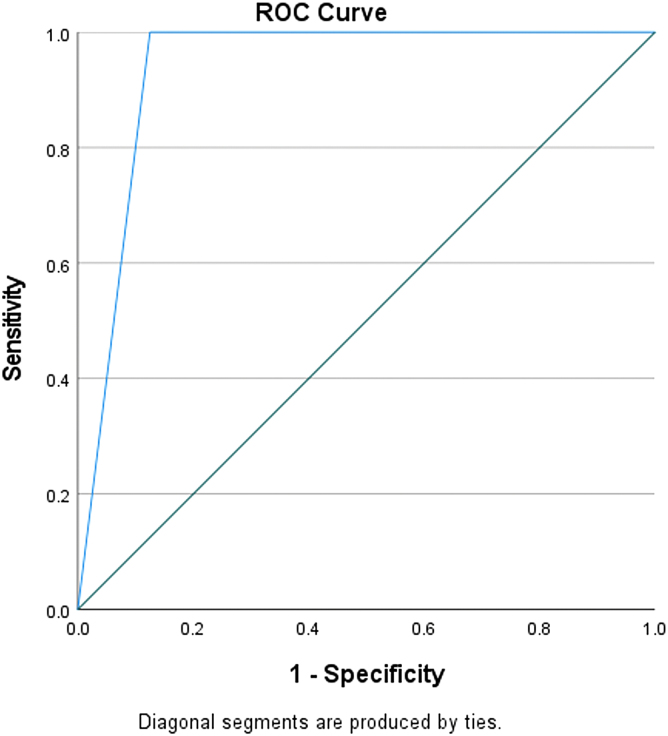
Receiver operating characteristic (ROC) curve of IL-5 concentrations to discriminate between cSDH patients with hematoma thickness >20 mm. IL-5, interleukin 5; cSDH, chronic subdural hematoma; mm, millimeter.

**FIG. 6.**
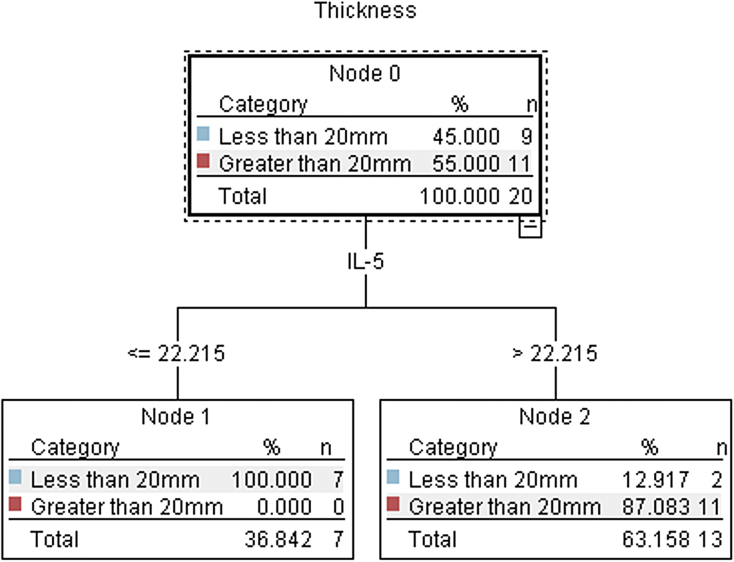
C5.1 decision tree demonstrating that IL-5 concentrations >22.215 pg/mL were significantly associated with hematoma thickness >20 mm in cSDH patients. IL-5, interleukin 5; mm, millimeter; cSDH, chronic subdural hematoma; pg, picogram; mL, milliliter.

The C5.1 algorithm built a decision tree model with 90% accuracy and an AUC = 0.94 (Fig. 5). The only significant predictor of hematoma thickness >20 mm was an IL-5 concentration of 22.22 pg/mL (Fig. 6). An IL-5 concentration above that value was associated with an RR of 4.5 (95% CI, 1.3–15.3; *p* < 0.001) compared to patients with an IL-5 concentration below that value. When using the IL-5 cutoff as a predictor in a univariate logistic regression model of hematoma thickness >20 mm, it accounted for 85% of the variance (*R*^2^ = 0.85, *p* < 0.001). cSDH thickness >20 mm on the presenting CT scan has been found to be an independent predictor of cSDH recurrence.

#### Predicting recurrence

**FIG. 7.**
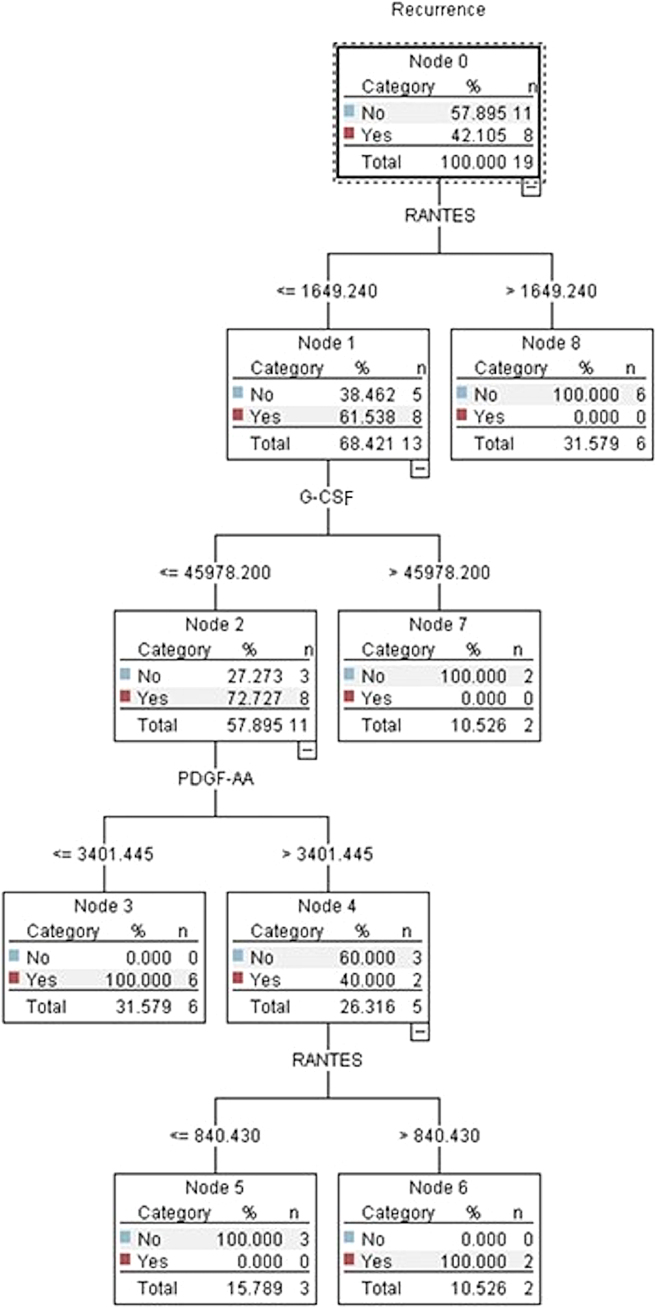
C5.1 decision tree demonstrating that inflammatory biomarker concentrations (pg/mL) were significantly associated with cSDH recurrence. RANTES, regulated on activation, normal T-cell expressed and secreted; G-CSF, granulocyte colony-stimulating factor; PDGF-AA, platelet-derived growth factor-AA; pg, picogram; mL, milliliter.

The C5.1 algorithm built a decision tree model with 95% accuracy and an AUC = 1.0. The algorithm identified the RANTES concentration of 1649.24 as the strongest differentiator of cSDH recurrence (Fig. 7), such that a RANTES concentration <1649.24 pg/mL was associated with an RR of 2.2 (95% CI, 1.15–4.20; *p* = 0.01) compared to patients with a RANTES concentration above the cutoff. When using the RANTES cutoff as a predictor in a logistic regression model of recurrence, it accounted for 49% of the variance (*R*^2^ = 0.49, *p* < 0.001).

Two other blood biomarkers were included in the decision tree model. Among patients with lower RANTES values, G-CSF concentrations ≤45,978.2 pg/mL had recurrence at a 73% rate compared to the patients with higher G-CSF values (0% recurrence rate). Among patients with lower RANTES and lower G-CSF values, patients with PDGF-AA concentrations ≤3401.45 pg/mL had recurrence at a 100% rate compared to patients above the PDGF-AA cutoff (40% recurrence rate).

## Discussion

As cSDH increases in prevalence in an aging population, concern of its socioeconomic burden on the elderly and the healthcare system grows.^1–13^ Further, cSDH can go unnoticed clinically, given that symptoms become more detectable over the course of weeks.^27,28^ Notably, the 20 patients included in the current study all presented with a GCS score of 15. Mild symptomology can lead to CT scans not being utilized. Advances in clinical management inclusive of predictive models are a necessity for improved diagnosis, treatment, and detection of recurrence. Increased feasibility of peripheral and local biomarkers as a supplement to clinical management stems from recent advancements in biomarker analysis technology, setting the stage for their usage in a predictive model.^29–31^ Inflammatory markers CXCL9, IL-6, VEGF-A, IL-5, and RANTES correlate with neurological outcome, cSDH thickness, MLS, and complete resolution of cSDH after evacuation. CXCL9 was predictive of MLS, IL-5 was predictive of cSDH thickness, and RANTES was predictive of cSDH recurrence. Our results mark these five biomarkers as potential candidates for a preliminary model that is not yet suitable for clinical use.

### Role of biomarkers in chronic subdural hematoma development

CXCL9, also known as monokine induced by gamma interferon, participates in the inflammatory response through promotion of chemotaxis and differentiation of leukocytes, inhibition of angiogenesis, and tissue extravasation.^32^ An animal model study found that CXCL9 promotes neural differentiation in neural precursor cells, which respond to central nervous system injury, as well as the maturation of oligodendrocytes.^33^ Stanisic and colleagues found that CXCL9 levels in cSDH fluid were significantly greater than levels in serum, suggesting a local mechanism confined to the hematoma area acting as an impetus to cSDH growth.^34^ This is consistent with the present study that higher CXCL9 levels are correlated with greater cSDH thickness and predictive of MLS, which may be attributable to its role in a local mechanism of angiogenesis inhibition and tissue extravasation. CXCL9 has already been proven as a viable biomarker in cardiac disease,^35,36^ bolstering the case for its use in a predictive cSDH model.

Similarly, IL-5 is a proinflammatory cytokine that acts as a key mediator in the growth, migration, and activation of eosinophils.^37,38^ Results from a mouse model study suggest that IL-5 plays a role in the interplay between immune cells and brain cells,^39^ whereas another rodent model of blast neurotrauma exhibited elevated levels of IL-5 in various parts of the brain, suggesting that IL-5 may be a key inflammatory mediator after blast injury.^40^ Further, studies have found elevated IL-5 levels in cSDH compared to cerebrospinal fluid (CSF) and venous blood.^41,42^ Eosinophils play a conductive role in the development of cSDH inflammation and membranes,^43^ with data from one study displaying a statistically significant correlation between IL-5 and eosinophil-derived neurotoxin (EDN), and EDN with the percentage of eosinophils in cSDH fluid.^41^ This supports the present findings, that increased IL-5 can be predictive of hematoma thickness.

IL-6 is a proinflammatory cytokine that plays a key role in the host defense response to infection or tissue damage by catalyzing the acute immune response and hematopoiesis.^44^ Further, IL-6 aids in the shift from acute to chronic inflammation.^45^ In the brain, IL-6 fosters the synthesis of nerve growth factor, as well as the survival and differentiation of neurons, with evidence suggesting its expression to be favorable post-injury.^46–49^ Whereas levels of IL-6 in CSF remain low under homeostatic conditions, they have been shown to spike post-TBI.^50,51^ In cSDH patients, Suzuki and colleagues found significantly greater concentrations of IL-6 in subdural fluid than in blood samples.^52^ Moreover, Frati and colleagues found significantly higher levels of IL-6 in subdural fluid in patients who presented with cSDH recurrence than patients without recurrence.^53^ These previous studies are consistent with our findings that higher IL-6 levels correlate with greater MLS, which may represent a dose-response relationship with cSDH severity, suggesting its potential role as a predictive biomarker.

### Risk of chronic subdural hematoma recurrence after surgery

VEGF-A, also simply called VEGF, is the chief moderator of blood vessel formation, playing a key role in the inflammatory process through angiogenesis, vasculogenesis, hematopoiesis, and by increasing vascular permeability.^54–56^ In the brain, VEGF additionally acts to moderate neuroprotection and neural migration; however, excessive concentrations can lead to edema through increased leakage across intracellular barriers and has also been linked to neurological disorders such as ischemic stroke.^57^ Immunohistochemical findings exhibit substantial expression of VEGF in inflammatory cells permeating cSDH neomembranes.^58^ Similar to IL-6, studies reported VEGF to have greater concentrations in subdural fluid than in serum.^58–60^ Though VEGF is an important contributor of cSDH development, as previously described,^61^ findings from a recent study suggest that VEGF is not a reliable predictive biomarker for cSDH recurrence.^62^ Despite our findings indicating a correlation between VEGF concentration and greater MLS, further studies are required to determine its potential as a biomarker predictive of cSDH recurrence.

The current study found a positive correlation between higher levels of RANTES with complete resolution of cSDH after evacuation, and our decision tree model found RANTES to be predictive of cSDH recurrence, with concentrations <1649.24 pg/mL associated with a greatly increased risk of recurrence. This is an interesting finding, given that a previous study reported a positive association between RANTES and cSDH recurrence requiring reoperation.^63^ Although RANTES is associated with chronic inflammation and might be a factor in angiogenesis,^64^ studies indicate that RANTES has a neuroprotective role and increases neuronal survival.^65,66^ Further investigation is required to fully elucidate the role of RANTES in cSDH recurrence.

### Limitations

Our findings are limited by the sample size, yet we intend to broaden the study population as our research continues. As such, the decision trees presented should not be considered suitable for clinical use at this time. Further, several analyte data values were absent from our current analysis because of being outside the Luminex assay's range of sensitivity. Future studies should incorporate least absolute shrinkage and selection operator regression in order to maximize usage of the collected analyte data. The scope of the current study focused on subdural fluid; however, analyzing analyte concentrations in plasma and serum are part of our future directives. We aimed our pilot investigation at the analytes that met a minimum mean threshold of 1000 pg/mL; however, future directives should contrarily examine analytes below a maximum mean threshold to determine whether any analytes are being suppressed in cSDH compared to baseline.

## Conclusion

This pilot study is the first analysis to date to compare analyte levels from operative cSDH fluid with neurological outcomes. This analysis identified five biomarkers (CXCL9, IL-6, VEGF-A, IL-5, and RANTES) as possible biomarkers for predicting cSDH characteristics, recurrence, and neurological outcomes. These preliminary results suggest the need for a larger study for validation and comparative analyses in peripheral plasma and/or serum biospecimens. These analytes play specific roles in proinflammatory response and angiogenesis, elevated levels of which can be associated not only with larger initial cSDH and mass effect, but also higher risk of recurrence after surgical evacuation. Future analyses could allow for a predictive, peripheral surrogate marker for cSDH reoccurrence and radiological imaging.

## Supplementary Material

Supplemental data

## Abbreviations Used

AUC: area under the curve
BTRC: Brain Trauma Research Center
CI: confidence interval
cSDH: chronic subdural hematoma
CSF: cerebrospinal fluid
CT: computed tomography
CXCL9: chemokine (C-X-C motif) ligand 9
EDN: eosinophil-derived neurotoxin
GCS: Glasgow Coma Scale
G-CSF: granulocyte colony-stimulating factor
GOS-E: Glasgow Outcome Scale-Extended
IL-1α: interleukin 1 alpha
IL-1β: interleukin 1 beta
IL-1Ra: interleukin 1 receptor antagonist
IL-5: interleukin 5
IL-6: interleukin 6
IL-8: interleukin 8
IL-10: interleukin 10
IP-10: interferon-γ-inducible protein 10
MCP-1: monocyte chemoattractant protein-1
M-CSF: macrophage colony-stimulating factor
MDC: macrophage-derived chemokine
MLS: midline shift
MMA: middle meningeal artery
MRI: magnetic resonance imaging
N/A: not applicable
PDGF-AA: platelet-derived growth factor-AA
PDGF-AB BB: platelet-derived growth factor-AB,-BB
RANTES: regulated on activation, normal T-cell expressed and secreted
RR: relative risk
SD: standard deviation
T1W1: T1-weighted image
TBI: traumatic brain injury
VEGF: vascular endothelial growth factor
