# Supplementary material for: Pilot Biomarker Analysis and Decision Tree Algorithm Modeling of Patients with Chronic Subdural Hematomas
Source: Neurotrauma Rep. 2023 Mar 24;4(1):184–96. doi: 10.1089/neur.2022.0062 (PMC10039273; doi:10.1089/neur.2022.0062)
Supplement: Supplemental data [file Suppl_TableS1.docx]

**Running Title:** Biomarker Analysis of Chronic Subdural Hematomas

Supplemental Table 1. Complete Mean Analyte Data

| **Analyte** | **Mean (SD) (pg/ml)** |
| --- | --- |
| CXCL9 | 16484.76 (19752.39) |
| MCP-1 | 12291.55 (5422.59) |
| G-CSF | 11734.69 (18225.92) |
| IL-6 | 6967.71 (3969.95) |
| VEGF-A | 5166.21 (4538.88) |
| IL-8 | 4206.02 (2834.98) |
| PDGF-AB BB | 4011.25 (3934.01) |
| PDGF-AA | 3654.59 (2578.93) |
| MDC | 2693.38 (2026.49) |
| IL-1α | 2228.97 (6429.97) |
| IP-10 | 2029.45 (2968.25) |
| RANTES | 1827.40 (3234.22) |
| IL-10 | 1800.45 (5418.43) |
| IL-1β | 1588.65 (5535.27) |
| IL-1Ra | 1518.49 (4179.95) |
| IL-5 | 1359.4 (3041.09) |
| M-CSF | 1067.18 (1139.76) |
| GRO-α | 933.96 (1154.55) |
| MCP-3 | 752.64 (1056.75) |
| FGF-2 | 593.73 (1063.21) |
| IL-27 | 591.95 (513.99) |
| GM-CSF | 558.23 (1822.58) |
| IL-25 | 556.60 (178.37) |
| IL-13 | 447.75 (942.95) |
| TNF-α | 329.58 (560.05) |
| IL-12 | 297.41 (464.25) |
| IL-18 | 208.53 (324.84) |
| MIP-1α | 158.25 (388.11) |
| IL-17F | 155.57 (245.35) |
| FLT-3L | 105.15 (61.25) |
| sCD40L | 103.03 (43.06) |
| IL-22 | 96.91 (41.32) |
| Fractalkine | 84.04 (37.55) |
| MIP-1β | 77.41 (121.56) |
| TNF-β | 65.71 (38.12) |
| Eotaxin | 64.98 (56.28) |
| IFN-γ | 46.48 (111.54) |
| IL-9 | 39.48 (58.19) |
| TGF-α | 27.12 (53.68) |
| IFNA2 | 22.54 (12.93) |
| EGF | 20.86 (23.50) |
| IL-15 | 20.52 (15.17) |
| IL-4 | 11.61 (7.22) |
| IL-17A | 7.90 (4.97) |
| IL-12 | 7.28 (3.46) |
| IL-2 | 3.38 (4.18) |
| IL-7 | 2.27 (1.67) |
| IL-3 | 0.65 (0.44) |

Supplemental Table 1. Complete mean analyte data.

SD, standard deviation; pg, picogram; ml, milliliter; CXCL9, chemokine (C-X-C motif) ligand 9; MCP-1, monocyte chemoattractant protein-1; G-CSF, granulocyte colony-stimulating factor; IL-6, interleukin 6; VEGF-A, vascular endothelial growth factor; IL-8, interleukin 8; PDGF-AB BB, platelet-derived growth factor-AB,-BB; PDGF-AA, platelet-derived growth factor-AA; MDC, macrophage-derived chemokine; IL-1α, interleukin 1 alpha; IP-10, interferon-γ-inducible protein 10; RANTES, Regulated on Activation, Normal T Cell Expressed and Secreted; IL-10, interleukin 10; IL-1β, interleukin 1 beta; IL-1Ra, interleukin-1 receptor antagonist; IL-5, interleukin 5; M-CSF, macrophage colony-stimulating factor; GRO-α, growth-regulated oncogene alpha; MCP-3, monocyte chemotactic protein-3; FGF-2, fibroblast growth factor 2; IL-27, interleukin 27; GM-CSF, granulocyte-macrophage colony-stimulating factor; IL-25, interleukin 25; IL-13, interleukin 13; TNF-α, tumor necrosis factor alpha; IL-12, interleukin 12; IL-18, interleukin 18; MIP-1α, macrophage inflammatory protein 1 alpha; IL-17F, interleukin 17F; FLT-3L, FMS-related tyrosine kinase 3 ligand; sCD40L, soluble CD40 ligand; IL-22, interleukin 22; MIP-1β, macrophage inflammatory protein 1 beta; TNF-β, tumor necrosis factor beta; IFN-γ, interferon gamma; IL-9, interleukin 9; TGF-α, transforming growth factor alpha; IFNA2, interferon alpha 2; EGF, epidermal growth factor; IL-15, interleukin 15; IL-4, interleukin 4; IL-17A, interleukin 17A; IL-12, interleukin 12; IL-2, interleukin 2; IL-7, interleukin 7; IL-3, interleukin 3.
